# Tonoplast-Localized Theanine Transporter CsCAT2 May Mediate Theanine Storage in the Root of Tea Plants (*Camellia sinensis* L.)

**DOI:** 10.3389/fpls.2021.797854

**Published:** 2021-12-17

**Authors:** Lin Feng, Yongchao Yu, Shijia Lin, Tianyuan Yang, Qi Chen, Linlin Liu, Jun Sun, Pengcheng Zheng, Zhaoliang Zhang, Xiaochun Wan

**Affiliations:** ^1^State Key Laboratory of Tea Biology and Resource Utilization, School of Tea and Food science and Technology, Anhui Agricutural University, Hefei, China; ^2^Institute of Fruit and Tea, Hubei Academy of Agricultural Sciences, Wuhan, China

**Keywords:** *Camellia sinensis* (L.) O. Kuntze, amino acid transporter, theanine storage, root-to-shoot transport, amino acid

## Abstract

Theanine is the component endowing tea infusion with “umami” taste and antidepression benefits. Theanine is primarily synthesized and stored in root in winter and is transported *via* vascular tissues to the new shoot in spring. However, the mechanism underlying theanine storage in the root of tea plants remains largely unknown. Cationic amino acid transporter 2 (CsCAT2) in tea plants is homologous to glutamine permease 1 (GNP1), the specific glutamine transporter in yeast. In this study, we identified CsCAT2 as an H^+^-dependent theanine transporter with medium affinity for theanine. The result of subcellular localization showed that CsCAT2 was a tonoplast-localized transporter. Importantly, *CsCAT2* highly expressed in the root in winter during theanine storage and reduced its expression in the root during theanine transport from root-to-shoot in spring. In addition, *CsCAT2* expression in the roots of 5 varieties at four time points during December and April was significant negatively correlated with the capacity of theanine root-to-shoot movement. Taken together, these results suggested that CsCAT2 may mediate theanine storage in the vacuole of root cells and may negatively modulate theanine transport from root to shoot.

## Introduction

Tea plant (*Camellia sinensis* L.) is a perennial and commercially valuable crop; its buds and one or two leaves below the bud are harvested for green tea manufacturing. The composition and content of amino acids in the tender shoot leaves are the critical components that endow tea with its flavor and health benefits (Wan, 2003; Hunt et al., 2010; Wan and Xia, 2015). Most amino acids contribute to the umami taste and alleviate the bitterness and astringency of tea infusions. The ratio of amino acids to polyphenols determines the quality and suitability of tea (Wan, 2003).

In the tender tea leaves, theanine accounts for more than 50% of the free amino acid content, which significantly correlates with the quality and price of green teas (Yamaguchi and Ninomiya, 2000; Wan, 2003; Feng et al., 2014). Theanine-derived metabolites produced during manufacturing contribute a roasted peanut flavor to oolong teas (Guo et al., 2018) and a roasted and caramel flavor to yellow tea (Guo et al., 2019). Theanine also contributes to the colors of tea infusion (Yao et al., 2006), given that the colored compounds containing theanine-carbohydrate conjugates (Tanaka et al., 2005). Theanine also has many health benefits including promoting relaxation, concentration, and learning efficiency (Sharma et al., 2018).

Theanine is mainly synthesized, stored in the roots in late autumn and winter, and transported to the new shoots in spring (Ruan et al., 2012; Ashihara, 2015). However, theanine metabolism and transport processes are weak in summer and fall (Gong et al., 2020). Therefore, generally, green teas produced in spring contain higher level of theanine than those produced summer and fall (Xu et al., 2012; Jiang et al., 2018). That is the main reason why green teas produced in spring are of high quality and price (Xu et al., 2012).

In tea plant roots, theanine is mainly synthesized from ethylamine and glutamate by theanine synthetase (CsTSI) (Wei et al., 2018; Fu et al., 2021; Zhu et al., 2021). *CsTSI* is highly and specifically expressed in roots and encodes an enzyme localized in cytoplasm, suggesting that theanine is synthesized in cytoplasm of root cells (Fu et al., 2021). Fu et al. (2021) examined the subcellular distribution of theanine and found almost all the theanine (~99%) was in the cytoplasm of root cells in March. However, in shoot, theanine was shown to be distributed in chloroplast, vacuole, cytoplasm, and mitochondria; the distribution was also dynamically changed within March, May, and November, and in response to shading treatment. Given that theanine is stored in root cells in winter and is transported to new shoots in spring, its subcellular distribution in root cells is also supposed to be dynamic. However, the dynamic subcellular distribution of theanine in roots in winter and spring has not been revealed.

Amino acid transporters (AATs) are membrane-localized proteins that mediate intercellular, intracellular, and long-distance amino acid transport (Tegeder et al., 2007; Dinkeloo et al., 2018). Recently, we identified amino acid permease (AAP) family members as theanine transporters in tea plants (Dong et al., 2020). Our results revealed that six CsAAPs exhibited theanine transport activity and are mainly expressed in the leaves, vascular bundles, and roots. Furthermore, *CsAAP1* is crucial in mediating the long-distance root-to-shoot transport of theanine (Dong et al., 2020). However, there is no clue implying that CsAAPs play a role in theanine storage in root.

In the abovementioned study, we found that glutamine permease 1 (GNP1) in yeast can also transport theanine. We searched for GNP1 homolog in tea plant by high sequence similarity. But there was no highly conserved GNP1 homolog found. The most closed protein in tea plant is CsCAT2 (Tea025016.1), which has 26.2% sequence identity to GNP1. Interestingly, in another study, we found *CsCAT2* expression in roots of tea plants was significantly induced by theanine feeding and was also induced by cold stress (Feng et al., 2018). These clues led us to ask whether CsCAT2 has theanine transport activity and plays a role in theanine transport in tea plants.

In this study, the substrate specificity and pH-dependence of CsCAT2 theanine transport were analyzed in yeast mutant 22Δ10α. The seasonal regulation of CsCAT2 expression in tea plants was analyzed, and correlation analysis was also conducted to elucidate whether CsCAT2 is involved in theanine storage and long-distance transport. Our findings indicated that CsCAT2 is a tonoplast-localized theanine transporter and its expression in the root was highly and negatively correlated with theanine accumulation in the leaf buds. These results elucidate CsCAT2's putative function in theanine storage in the root of in tea plants.

## Materials and Methods

### Transient Expression in Protoplasts

The full cDNA sequence of *CsCAT2* without the stop codon was cloned into a pK7WGF 7.0 vector carrying the 35S promoter and green fluorescent protein. The plasmids encoding the fusion protein CsCAT2 were transiently transformed using chemical shock into protoplasts derived from an *Arabidopsis* cell suspension culture. Two days after the polyethylene glycol-mediated plasmid transformation, these protoplasts were analyzed using confocal microscopy (FV1000, Olympus, Japan).

### Total RNA Extraction and Quantitative RT-PCR

Tea plants used were planted in Guohe Tea Plantation (Lujiang, Anhui, China). For season-dependent *CsCAT2* expression analysis, total RNA was extracted from the roots of five tea plant varieties (Zhenong 113, Yingshuang, Zhonghuang 302, Longjing 43, and Shuchazao) collected on December 12, 2017 and March 1, March 23, and April 13, 2018. The real-time reverse transcription quantitative PCR procedures and analysis were conducted following the method of Feng et al. (2018). Gene-specific primers were designed using Primer Premier 5 and was listed in Supplementary Table 1. Glyceraldehyde 3-phosphate dehydrogenase (*GAPDH*) gene (Song et al., 2016) was used as an internal control. Data were analyzed following the threshold cycle (Ct), and relative expression was quantified using 2^−ΔCt^ (Schmittgen and Zakrajsek, 2000).

### Cloning of CsCAT2 and Yeast Complementation Assay

The sequences of CsCAT2 were amplified using the polymerase chain reaction (PCR) from Shuchazao leaves cDNA libraries and cloned with XbaI in a pYES2 yeast expression vector. The primers used in the study were presented in Supplementary Table 1. Recombinant plasmid or pYES2 were transferred into the 22Δ10α yeast strain (this yeast strain does not have 10 AATs and cannot grow on medium with amino acid as the sole nitrogen, except arginine) (Besnard et al., 2016), generating strains designated as 22Δ10α-*CsCAT2* and 22Δ10α-pYES2. Recombinant strains were selected on the yeast nitrogen base lacking uracil and were supplied with 2 mM ammonium sulfate. After the PCR verification, positive colonies were selected for further transport analysis.

### Growth Assays of CsCAT2 on Amino Acid as the Sole Nitrogen Source

Growth assays were conducted in uracil-free YNB medium containing 2 mM of theanine, glutamine, aspartic acid, asparagine, glutamic acid, proline, γ-aminobutyric acid, or citrulline as sole nitrogen source and 2% d-galactose as the carbon source. Yeast cultured to an OD_600_ of 0.6 was diluted to 10^0^, 10^−1^, 10^−2^, 10^−3^, or 10^−4^ solutions. However, 2 μl diluted solutions were spotted onto the YNB media. The yeast growth was observed after 3 days of incubation at 30°C and was photographed with a digital camera. All experiments were performed for three independent replicates.

For growth assays in a liquid medium, 0.2 and 2 mM of theanine were used to analyze the growth of yeast, and the OD_600_ values were measured daily over 7 days.

### Measurement of Theanine Transport

For kinetic analysis, cells of the wild-type yeast strains 23344c, 22Δ10α-pYES2, and 22Δ10α-CsCAT2 were grown on the YNB and 2 mM ammonium sulfate medium to OD_600_ = 0.8. Yeast cells were collected, washed three times, and then cultured in the YNB medium without nitrogen for 2 h of starvation. Theanine was added to varied concentrations of 0.09375, 0.1875, 0.375, 0.75, 2.25, and 8 mM. Yeast cells were centrifuged and collected at 0, 2, 5, 10, and 20 min after theanine was added. For the substrate specificity analysis, 200 μM of theanine was added to the respective solutions along with 2 mM of the each of the following competitors: valine, aspartic acid, alanine, glutamate (Glu), and glutamine (Gln). Yeast cells were collected and centrifuged for 10 min. To analyze the pH-dependence of theanine uptake after the nitrogen starvation treatment, the pH of the medium was adjusted to 4, 5, 6, 7, and 8 using hydrochloric acid or sodium hydroxide before the addition of 200 μM. Yeast cells were collected and centrifuged for 10 min. The pretreatment procedures for the H^+^ pump inhibitor treatment before and after theanine feeding were identical to those of the kinetic analysis; 200 μM of theanine was added along with 0.01 mM carbonylcyanide m-chlorophenylhydrazone (CCCP), 0.1 mM 2, 4-dinitrophenol (DNP), and 1 mM diethylpyrocarbonate (DEPC). Yeast cells were collected and centrifuged for 10 min.

The yeast cultivation and sample processing procedures of (a), (b), (c), and (d) were identical. Following the collection of the sediment, (a), (b), (c), and (d) were each collected and washed four times with pH 4.5 buffer, containing 0.6 M sorbitol and 50 mM potassium phosphate. After the addition of 1 ml of deionized water to the resuspended cells, the mix was bathed in 98°C water for 1 h for theanine extract.

Theanine was detected with a Waters HPLC system, equipped with a 2489 ultraviolet (UV)-visible detector and a reverse-phase C18 column (5 μm, 250 mm × 4.6 mm, Phenomenex, Los Angeles, USA). The column temperature and the detection wavelength were set to 28°C and 210 nm, respectively. The mobile phase consisted of HPLC H_2_O (A), acetonitrile (B), and the gradient elution was as follows: B 0% (v/v) to 80% at 12 min, to 0% at 22 min. The flow rate is 1 ml/min, and the injection volume is 10 μl. Then, theanine concertation was calculated according to the theanine standard.

### Statistical Analysis

Data were presented as mean ± standard deviation (SD) of three independent biological replicates. Statistical significance was evaluated through the one-way analysis of variance, followed by a Tukey's test using SPSS Statistics 19.0 (IBM, Chicago, USA). The correlation coefficient analysis was elucidated using the Pearson's correlation coefficient, with *p* < 0.05 was considered as statistical significance.

## Results

### CsCAT2 Localizes in the Tonoplast

**Figure 1:**
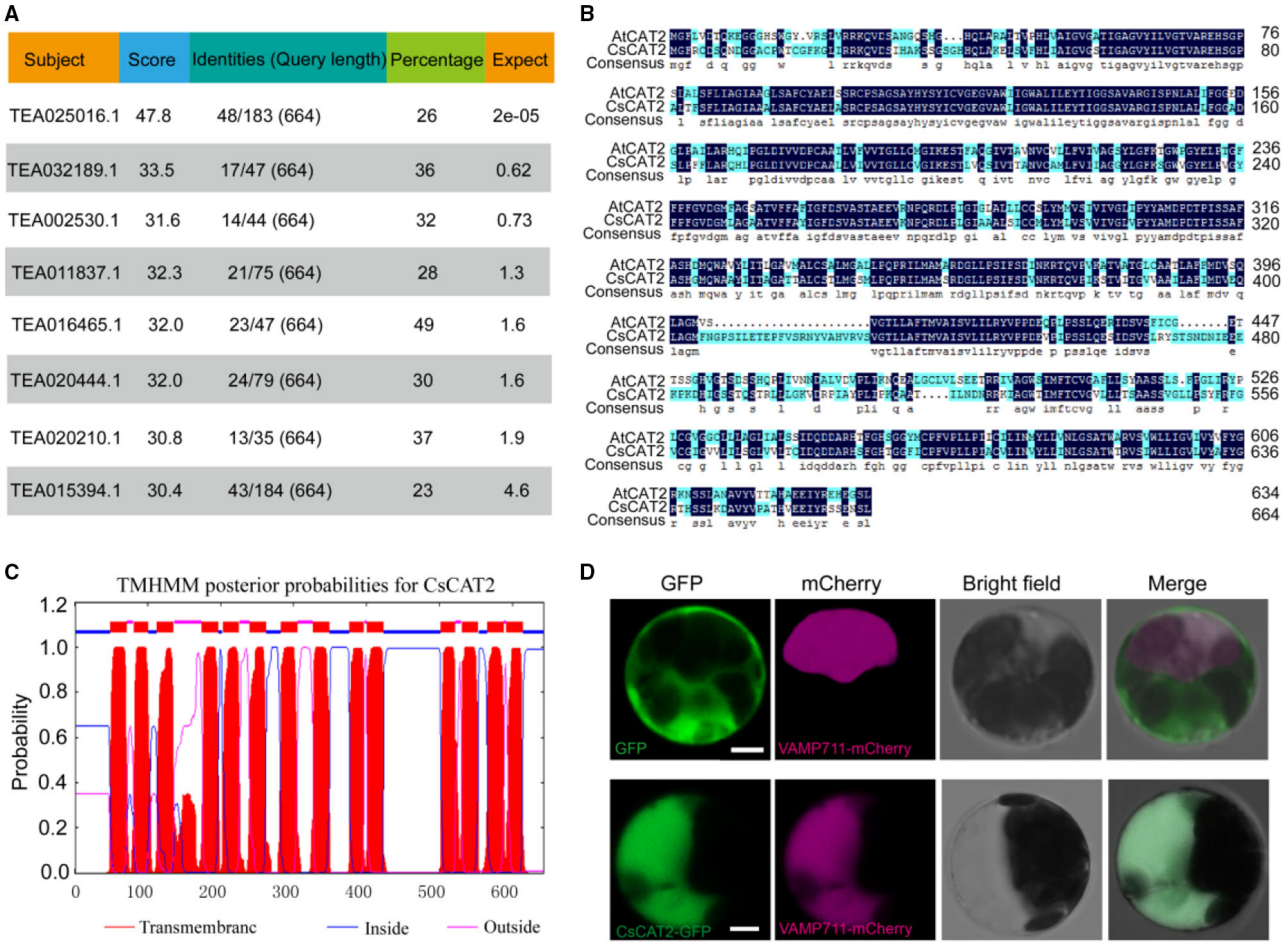
The sequence, transmembrane structure, and subcellular localization of CsCAT2. **(A)** The similarity of amino acid sequences of proteins in tea plant with the yeast theanine transporter GNP1 (YDR508C). **(B)** Sequence alignment of CsCAT2 and AtCAT2. **(C)** CsCAT2 transmembrane domain prediction. **(D)** Subcellular localization of CsCAT2. PK7WGF2.0-GFP and CsCAT2-GFP fusion proteins were expressed in *Arabidopsis* protoplasts. Bar = 5 μm.

Our previous study characterized that GNP1 in yeast has theanine transport activity (Dong et al., 2020). We tried to identify GNP1 homolog in tea plants, but we failed to find highly conserved homolog in tea plants. However, we found that a protein (TEA025016.1) in tea plant has weak conservation with GNP1 (Figure 1A). TEA025016.1 is highly homologous to AtCAT2 (Figure 1B), a tonoplast-localized AAT in *Arabidopsis*. Therefore, we named TEA025016.1 as CsCAT2. CsCAT2 is conserved in plants including *Arabidopsis thaliana, Vitis vinifera, Theobroma cacao, Actinidia* spp., *Populus tremula*, and *Coffea canephora* (Feng et al., 2018). CsCAT2 has also classic transmembrane domains based on the prediction using TMHMM online program (Figure 1C).

To verify the subcellular localization of CsCAT2, cauliflower mosaic virus 35S promoter-driven *CsCAT2*-*GFP* and *VAMP711*-*mCherry* were cotransferred into *Arabidopsis* protoplast. VAMP711 is a tonoplast-localized protein (Bassil et al., 2011) and was used as the tonoplast marker in this study. The fluorescence of CsCAT2-GFP overlapped with that of VAMP711-mCherry (Figure 1D), indicating that CsCAT2 also localized in the tonoplast.

### *CsCAT2* Expression Is Seasonally Regulated

**Figure 2:**
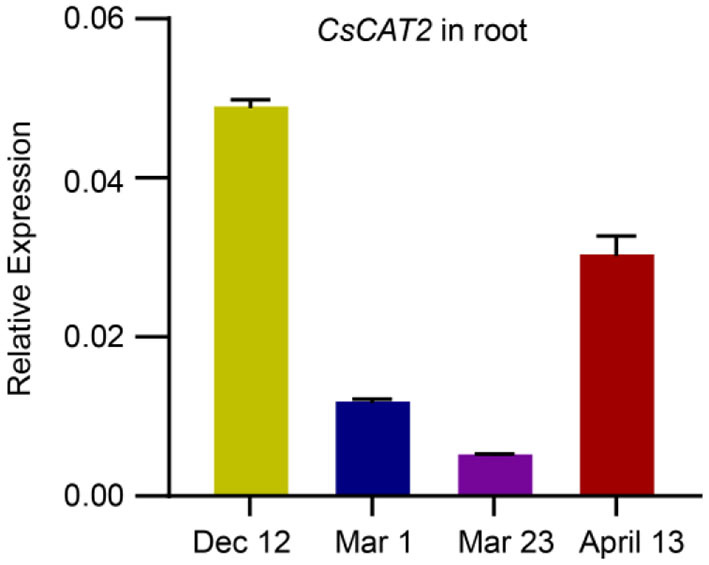
Seasonal expression of CsCAT2 in the root of tea plant cultivar “Shuchazao” tea cultivar. The expression of CsCAT2 in the root at four time points, December 12 (Dec 12), March 1 (Mar 1), March 23 (Mar 23), and April 13, was examined by qRT-PCR. The data were calculated using the 2^−Δct^ method and represented as mean ± SD (*n* = 3) with *CsGAPDH* as the internal control.

Theanine is mainly synthesized and stored in roots in winter and is then transported to new shoots in early spring (Ruan et al., 2012; Ashihara, 2015). To explore the role of CsCAT2 in theanine storage and root-to-shoot transport, we examined *CsCAT2* expression in roots at four time points including December 12, March 1, March 23, and April 13. *CsCAT2* expression was high at the December 12 time point, continuously decreased from December 12 to March 1 and March 23, and subsequently increased at April 13 (Figure 2). In contrast, theanine contents in new shoots continuously increased from December 1 to March 1 and March 23 and subsequently increased at April 13 (Dong et al., 2020). Additionally, theanine transport is strongest in March and then decreases in April (Fang et al., 2017; Dong et al., 2020). The consistency of *CsCAT2* expression with theanine storage in the roots suggested a role of CsCAT2 in theanine storage; the opposite change patterns between *CsCAT2* expression and theanine root-to-shoot transport suggested that CsCAT2 may negatively modulate theanine root-to-shoot transport.

### CsCAT2 Transports Theanine and Has a Medium Affinity for Theanine

**Figure 3:**
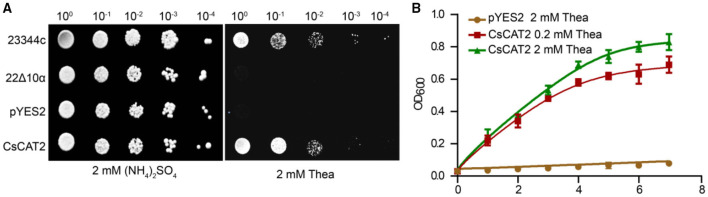
CsCAT2-mediated theanine uptake into yeast cells. **(A)** Dot assays showed the growth phenotype of yeast lines 23344c (wild-type line), mutants 22Δ10α, 22Δ10α/pYES2 (pYES2), and 22Δ10α/pYES2-*CsCAT2* (CsCAT2), on medium with 2 mM (NH_4_)_2_SO_4_ or 2 mM theanine (Thea) as nitrogen source. **(B)** The growth curve of yeast in liquid medium at different theanine concentrations as nitrogen source. The OD_600_ value was identified as a parameter for measuring yeast growth. 23344c and the empty vector pYES2 were the positive and negative controls, respectively. The result was expressed as mean ± SD (*n* = 3).

To explore the possibility that CsCAT2 regulates theanine storage and root-to-shoot transport, we asked whether CsCAT2 can directly transport theanine. We cloned the *CsCAT2* into a pYES2 vector and transformed it into a yeast mutant strain 22Δ10α. This mutant carries mutations of 10 genes encoding AATs and cannot grow on the medium with amino acid (except arginine) as the sole nitrogen source (Besnard et al., 2016). As shown in Figure 3, the wild-type strain 23344c grew well on the medium supplied with 5 mM theanine as the sole nitrogen source, whereas 22Δ10α strain and 22Δ10α that transferred with vector pYES2 could not grow. However, the expression of CsCAT2 in 22Δ10α conferred the growth of this mutant (Figure 3A). We further cultured these yeast strain 22Δ10α/pYES2 in liquid medium with 2 mM theanine, and 22Δ10α/CsCAT2 in liquid medium with 0.2 or 2 mM of theanine as the sole nitrogen source, to observe the growth. In this assay, we also observed 22Δ10α/CsCAT2 gradually grew during 10 days culture. But 22Δ10α/pYES2 did not grow (Figure 3B). These results indicated that CsCAT2 transported theanine into the yeast cells.

**Figure 4:**
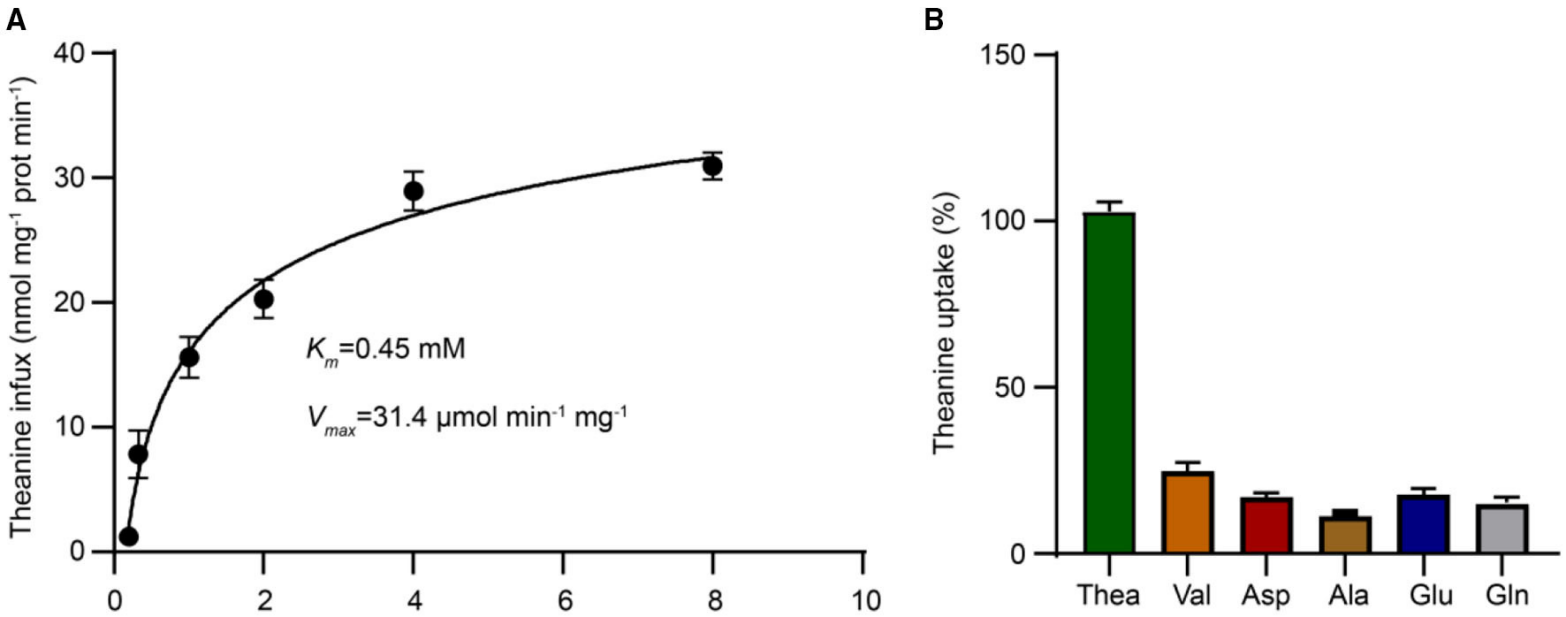
The uptake kinetic properties and substrate selectivity analysis of theanine transport by CsCAT2. **(A)** Measurement of *K*_*m*_ and *V*_*max*_ values of CsCAT2 for theanine in yeast. The ordinate represents the amount of theanine absorbed by per milligram protein per minute. Prot represents protein. **(B)** Substrate specificity of CsCAT2. Theanine uptake was determined with 10-dose excess glutamine (Gln), asparate (Asp), glutamate (Glu), alanine (Ala), and valine (Val), as the competitor, respectively. The group of uptake without competitive amino acid was served as 100%. The result was expressed as mean ± SD (*n* = 3).

To further assess the affinity of CsCAT2 for theanine, we next analyzed the transport kinetics of CsCAT2. The kinetic parameters calculated through theanine transport assays indicated that the *K*m and *V*_max_ for CsCAT2 were 0.45 mM and 31.43 μmol min^−1^ mg^−1^, respectively (Figure 4A). This suggested that CsCAT2 was a medium-affinity theanine transporter.

### CsCAT2 Has Broad Substrate Specificity

We next asked whether CsCAT2 can transport other amino acids. We cultured the above yeast strains in medium with Glu, glutamine (Gln), γ-aminobutyric acid (GABA), valine (Val), alanine (Ala), or aspartate (Asp), at a concentration of 2 mM, respectively, as the sole nitrogen source. The result demonstrated that CsCAT2 can also transport these amino acids (Supplementary Figure 1) and also demonstrated that CsCAT2 had broad substrate specificity, which was consistent with other AATs (Dong et al., 2020; Li et al., 2020, 2021).

To further investigate the substrate specificity of CsCAT2, a 10-fold dose of Val, Ala, Gln, Asp, and Glu, the dominant amino acids being transported in vascular system of tea plants (Ruan et al., 2012), was added to the medium to compete with theanine for transport. The results showed that CsCAT2's capacity to transport theanine was significantly suppressed by these amino acids (Figure 4B), further demonstrating that CsCAT2 can transport Glu, Gln, Ala, Asp, and Val. These results are consistent with other reports that CATs have broad substrate specificities and affinities for Glu, Ala, Gln, Asp, and Val in *Arabidopsis* (Su et al., 2004; Hammes et al., 2006; Yang et al., 2010, 2015).

### CsCAT2 Transports Theanine in a pH-Dependent Manner

**Figure 5:**
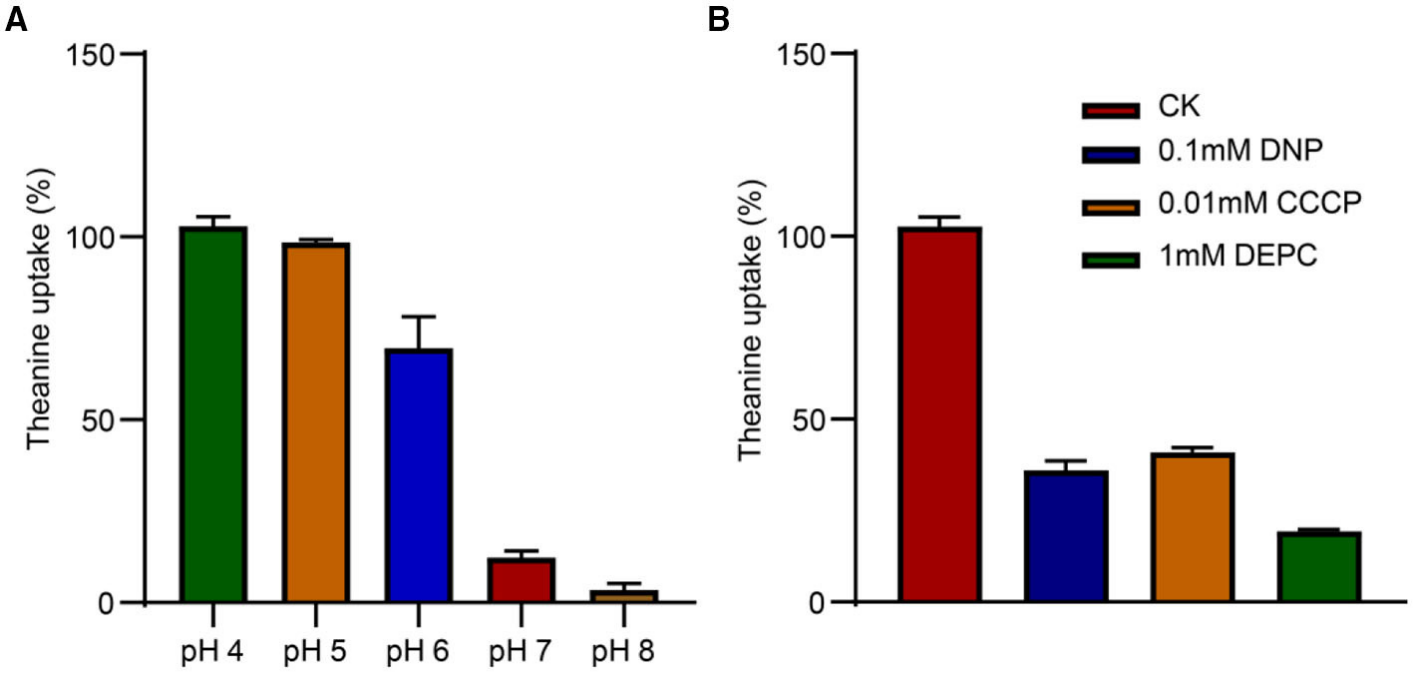
The pH dependence of CsCAT2 uptake of theanine in 22Δ10α. **(A)** Theanine uptake by CsCAT2 at different pH values; uptake at pH 4 serve as 100%. **(B)** Theanine uptake by CsCAT2 in the presence of CCCP, DNP, and DEPC. The result was expressed as mean ± SD (*n* = 3).

The transport capacity of theanine greatly decreased when the value of pH was increased from 4 to 8 in the medium (Figure 5A). At pH 7.0 and pH 8.0, theanine transport by CsCAT2 was almost completely inhibited. To further verify the pH or H^+^-dependant CsCAT2 activity, we added protonophores 2, 4-dinitrophenol (DNP) and carbonylcyanide m-chlorophenylhydrazone (CCCP), and an H^+^-coupled amino acid transport system inhibitor (DEPC), to the theanine transport system. The result showed that theanine transport activity of CsCAT2 was essentially inhibited by these reagents (Figure 5B). Therefore, CsCAT2 transport theanine is pH-dependant and functions as an H^+^-coupled theanine transport system.

### *CsCAT2* Expression in the Roots Probably Correlates With the Efficiency of Theanine Root-to-Shoot Transport

The efficiency of transport of theanine from root to shoot is critical for theanine accumulation in new shoots which are the materials for tea processing. As described (Figure 2), *CsCAT2* expression in the root showed an opposite trend with the process of theanine transport from root to shoot, at four time points (December 1, March 1, March 23, and April 13), in a tea plant cultivar “Shuchazao.” To further illustrate that CsCAT2 plays a role in theanine transport from root to shoot seasonally, we examined *CsCAT2* expression in the roots of five cultivars and analyzed the correlation between *CsCAT2* expression and the efficiency of theanine root-to-shoot transport.

Because theanine is primarily synthesized in the root in winter and then transported to the shoot, the ratio of theanine contents in the shoot and that in the root was used as a parameter for illustrating the efficiency of theanine transport from root to shoot (Dong et al., 2020). Moreover, theanine contents in the different tissues of shoot vary greatly and are highest in the leaf bud. Therefore, we used the sum of ratios of theanine contents in the roots and in the leaf buds at the four time points to quantify the efficiency of theanine root-to-shoot transport (Dong et al., 2020).

**Figure 6:**
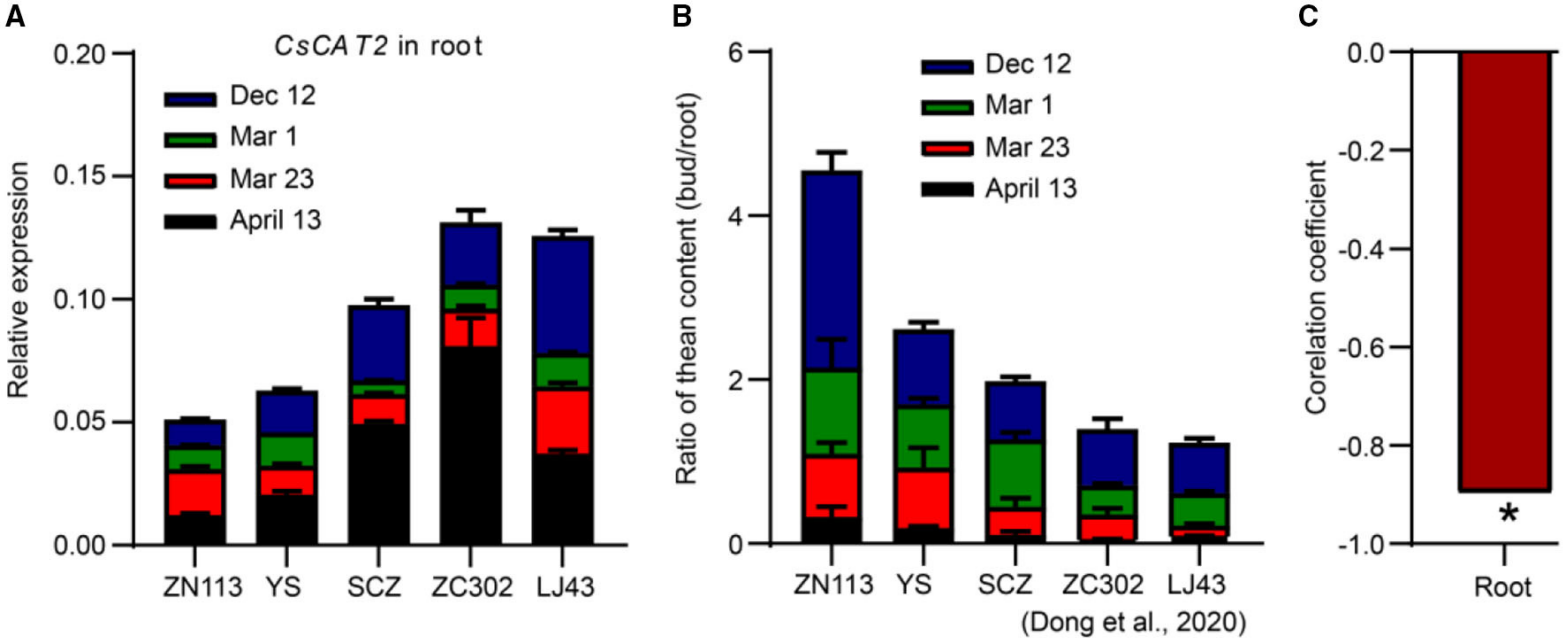
Correlation between *CsCAT2* expression in the roots and the efficiency of theanine root-to-shoot transport. **(A)** *CsCAT2* expression in the roots of 5 tea plant cultivars at the four time points. *CsGAPDH* was used as an internal control. **(B)** The ratios of theanine in the bud to that in the root in different tea plant cultivars. The data are from Dong et al. (2020). **(C)** Correlation between the summation of *CsCAT2* expression **(B)** and the ratios **(C)** was analyzed using Pearson's correlation. ZN113, Zhenong 113; YS, Yingshuang; SCZ, Shuchazao; ZC302, Zhongcha 302; LJ43, Longjing 43. Data were analyzed using one-way ANOVA, * *p* < 0.05.

Here, the sum of *CsCAT2* expression level in the root at different time points was calculated (Figure 6A). We observed that the sum was lowest in cultivar Zhenong 113 (ZN113) and gradually increased in Yingshuang (YS), Zhongcha 302 (ZC302), and Longjing 43 (LJ43). In contrast, the efficiency of theanine root-to-shoot transport was highest in ZN113 and gradually decreased in YS, ZC302, and LJ43 (Figure 6B) (Dong et al., 2020). More importantly, the sum of *CsCAT2* expression in the root was significant negatively correlated with the efficiency of theanine root-to-shoot transport, with a correlation coefficient of −0.897 (*p* < 0.05; Figure 6C). These results further supported the notion that CsCAT2 plays a negative role in theanine root-to-shoot transport.

## Discussion

### CsCAT2 Is a Tonoplast-Localized Theanine Transporter

**Figure 7:**
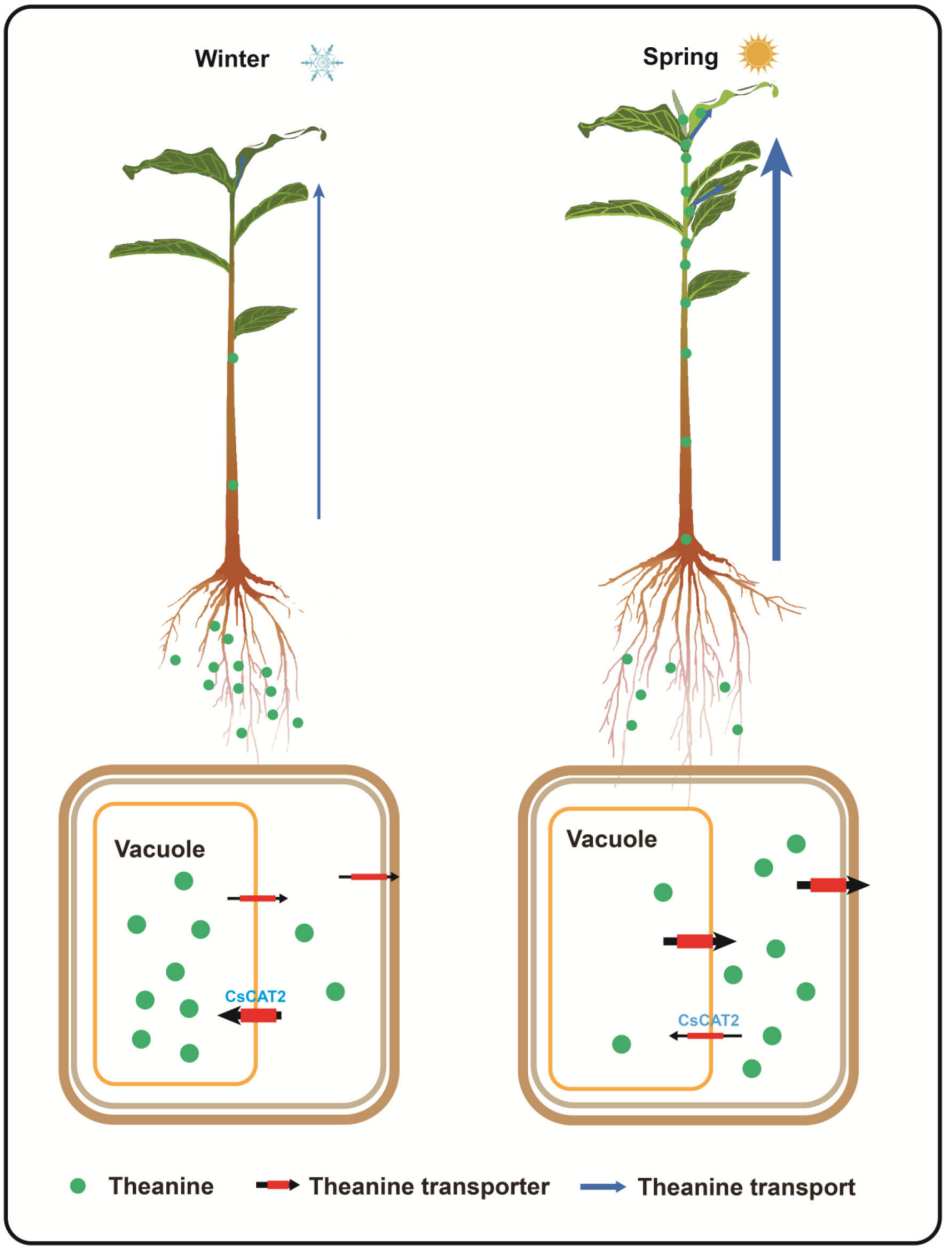
Proposed model for theanine storage and root-to-shoot transport in tea plants. In winter, theanine is transported into vacuole for storage; in spring, theanine is exported from vacuole into cytoplasm and is then transported out of the storage cells for root-to-shoot transport. CsCAT2 likely mediates theanine transport into the vacuole. High expression of *CsCAT2* in winter confers efficient transport of theanine into vacuole; however, high expression of *CsCAT2* in spring reduces the efficiency of theanine root-to-shoot transport.

In tea plants, theanine is primarily synthesized and stored in root cells, in late autumn and winter, and is transported to the new shoot, in spring (Gong et al., 2020). However, the molecular mechanism of theanine storage in root cells is still unknown. We hypothesized that theanine is stored in the vacuole and the storage is mediated and seasonally regulated by tonoplast-localized theanine transporters (Figure 7).

Several CAT families of AATs in plants have been shown to localize in the tonoplast and play important roles in amino acid homeostasis (Yang et al., 2013, 2014; Snowden et al., 2014). In *Arabidopsis*, AtCAT2 was shown to localize in the tonoplast and regulate amino acid levels in the leaves (Yang et al., 2014). SlCAT2, the homolog of CAT2 in tomato, was demonstrated to localize in the tonoplast of stamen cells and play a significant role in tomato flower and fruit development (Yang et al., 2013). SlCAT9, a relatively specific Glu/Asp/GABA transporter, regulates the accumulation of amino acids during fruit development and the flavor profile of the tomato fruit and has also been demonstrated to localize in the tonoplast (Snowden et al., 2014). In this study, we showed that CsCAT2 is a tonoplast-localized AAT (Figure 1).

Generally, CATs display board substrate specificity for neutral and acidic amino acids in plants (Su et al., 2004; Hammes et al., 2006; Yang et al., 2010, 2013, 2014, 2015; Snowden et al., 2014; Yoneda, 2017). In this study, we revealed that CsCAT2 is a theanine transporter and also has affinities for Glu, Gln, GABA, and Ala (Figures 3, 4). It is noteworthy that theanine contents in root cells are very high and could reach 60 mg/g dry weight (Li et al., 2019), whereas the contents of Glu, Gln, GABA, and Ala are much lower in root cells of tea plants (Yang et al., 2020). Therefore, CsCAT2 may mainly transport theanine even though it has affinities for other amino acids.

Interestingly, *CsCAT2* expression is high in winter, decreases in early spring, and then increases in late spring (Figure 6). This expression pattern is consistent with theanine storage and is opposite to theanine root-to-shoot transport. We further showed that *CsCAT2* expression was significantly correlated with the efficiency of theanine root-to-shoot transport (Figure 7). These findings supported the notion that CsCAT2 mediates theanine storage in vacuole in the root cells. In addition, theanine was shown to mainly distribute in the cytoplasm in March (Fu et al., 2021), which is possibly caused by greatly reduced level of CsCAT2 in the tonoplast (Figure 7). The cytoplasm distributed theanine in root cells is probably ready for root-to-shoot transport (Figure 7).

### CsCAT2 May Coordinate Seasonal Theanine Allocation Between Root and Shoot

Amino acids seasonally store in the roots and transport to shoots are economically efficient for adjusting plant growth according to the environment and nutrition requirement (Zeng et al., 2020; Fu et al., 2021). In early spring, the soil temperature is low, so that the absorption capacity of nitrogen from the soil is weak and insufficient to meet the nitrogen demand for the growth of new shoots. The transport of amino acids stored in root cells to the new shoot could be the effective safeguard of nitrogen requirement for the growth of new shoots (Zeng et al., 2020). Theanine is the dominant form of nitrogen storage in roots of tea plants (Ruan et al., 2012). Seasonally regulated *CsCAT2* expression likely contributes to theanine allocation between root and shoot to support new shoot growth.

In most tea plant-growing area, the shoots of tea plants have several rounds of growth-dormancy cycles, except the winter dormancy, in a year (Hao et al., 2017). We speculated that the downregulation of *CsCAT2* in the early spring might contribute to theanine transport to shoots to support the first round of shoot growth, whereas the upregulation at the late spring is conducive to reduce theanine transport to the shoots and promote bud dormancy, thereby store nitrogen sources for the next round of shoot growth in summer. Given that high-quality green teas are generally processed from the first round of new shoot of tea plants, the new shoots grown in summer are rarely harvested for green tea processing, which causes a great waste of tea resources. It will be intriguing to breed tea plant cultivars with strong growth in spring and weak growth in summer, using *CsCAT2* as a molecular marker.

## Data Availability Statement

The original contributions presented in the study are included in the article/Supplementary Materials, further inquiries can be directed to the corresponding authors.

## Author Contributions

LF, XW, and ZZ conceived the study and designed the experiments. LF, YY, and SL performed the experiments. TY, QC, LL, JS, and PZ supervised the project and participated in processing the data. LF and ZZ wrote the manuscript. XW and ZZ finalized the manuscript. All authors read and approved the final version of the manuscript.

## Funding

This work was supported by grants from the China Postdoctoral Science Foundation (2018M632821), the Hubei Provincial Natural Science Foundation (2019CFB178), Collaborative Innovation Project of Universities in Anhui Province (GXXT-2020-080), the Hubei Innovative Postdoctoral Position (20192344), Natural Science Foundation of Hubei Academy of Agricultural Sciences (2021NKYJJ13), and the Earmarked Fund for China Agriculture Research System (CARS-23).

## Conflict of Interest

The authors declare that the research was conducted in the absence of any commercial or financial relationships that could be construed as a potential conflict of interest.

## Publisher's Note

All claims expressed in this article are solely those of the authors and do not necessarily represent those of their affiliated organizations, or those of the publisher, the editors and the reviewers. Any product that may be evaluated in this article, or claim that may be made by its manufacturer, is not guaranteed or endorsed by the publisher.

## Supplementary Material

Supplementary Figure 1 Growth of yeast strains on medium with amino acid as sole nitrogen source, with 0 nitrogen (-N) and 2 mM (NH_4_)_2_SO_4_ as negative and positive controls.

Supplementary Table 1 Primers used in the experiments.

## References

[B1] Ashihara H. (2015). Occurrence, biosynthesis and metabolism of theanine (γ-glutamyl-L-ethylamide) in plants: a comprehensive review. Nat. Prod. Commun. 10, 803–810. 10.1177/1934578X1501000525 26058162

[B2] Bassil E. Tajima H. Liang Y. C. Ohto M. A. Ushijima K. Nakano R.. (2011). The Arabidopsis Na+/H+ antiporters NHX1 and NHX2 control vacuolar pH and K+ homeostasis to regulate growth, flower development, and reproduction. Plant Cell. 23, 3482–3497. 10.1105/tpc.111.089581 21954467 PMC3203450

[B3] Besnard J. Pratelli R. Zhao C. Sonawala U. Collakova E. Pilot G.. (2016). UMAMIT14 is an amino acid exporter involved in phloem unloading in Arabidopsis roots. J. Exp. Bot. 67, 6385–6397. 10.1093/jxb/erw412 27856708 PMC5181585

[B4] Dinkeloo K. Boyd S. Pilot G. (2018). Update on amino acid transporter functions and on possible amino acid sensing mechanisms in plants. Semin. Cell Dev. Biol. 74, 105–113. 10.1016/j.semcdb.2017.07.010 28705659

[B5] Dong C. Li F. Yang T. Feng L. Zhang S. Li F.. (2020). Theanine transporters identified in tea plants (*Camellia sinensis* L.). Plant J. 101, 57–70. 10.1111/tpj.14517 31461558

[B6] Fang R. Redfern S. P. Kirkup D. Porter E. A. Kite G. C. Terry L. A.. (2017). Variation of theanine, phenolic, and methylxanthine compounds in 21 cultivars of *Camellia sinensis* harvested in different seasons. Food Chem. 220, 517–526. 10.1016/j.foodchem.2016.09.047 27855934

[B7] Feng L. Gao M. J. Hou R. Y. Hu X. Y. Zhang L. Wan X. C.. (2014). Determination of quality constituents in the young leaves of albino tea cultivars. Food Chem. 155, 98–104. 10.1016/j.foodchem.2014.01.044 24594160

[B8] Feng L. Yang T. Zhang Z. Li F. Chen Q. Sun J.. (2018). Identification and characterization of cationic amino acid transporters (CATs) in tea plant (Camellia sinensis). Plant Growth Regul. 84, 57–69. 10.1007/s10725-017-0321-0

[B9] Fu X. Liao Y. Cheng S. Xu X. Grierson D. Yang Z. (2021). Nonaqueous fractionation and overexpression of fluorescent tagged enzymes reveals the subcellular sites of L-theanine biosynthesis in tea. Plant Biotechnol J. 19, 98–108. 10.1111/pbi.13445 32643247 PMC7769230

[B10] Gong A. D. Lian S. B. Wu N. N. Zhou Y. J. Zhao S. Q. Zhang L. M.. (2020). Integrated transcriptomics and metabolomics analysis of catechins, caffeine and theanine biosynthesis in tea plant (*Camellia sinensis*) over the course of seasons. BMC Plant Biol. 20:294. 10.1186/s12870-020-02443-y 32600265 PMC7322862

[B11] Guo X. Ho C. Schwab W. Song C. Wan X. (2019). Aroma compositions of large-leaf yellow tea and potential effect of theanine on volatile formation in tea. Food Chem. 280, 73–82. 10.1016/j.foodchem.2018.12.066 30642509

[B12] Guo X. Song C. Ho C. Wan X. (2018). Contribution of L-theanine to the formation of 2, 5-dimethylpyrazine, a key roasted peanutty flavor in Oolong tea during manufacturing processes. Food Chem. 263, 18–28. 10.1016/j.foodchem.2018.04.117 29784304

[B13] Hammes U. Z. Nielsen E. Honaas L. A. Taylor C. G. Schachtman D. P. (2006). AtCAT6, a sink- tissue-localized transporter for essential amino acids in Arabidopsis. Plant J. 48, 414–426. 10.1111/j.1365-313X.2006.02880.x 17052324

[B14] Hao X. Yang Y. Yue C. Wang L. Horvath D. P. Wang X. (2017). Comprehensive transcriptome analyses reveal differential gene expression profiles of Camellia sinensis axillary buds para-, endo-, ecodormancy, and bud flush stages. Front. Plant Sci. 8:553. 10.3389/fpls.2017.00553 28458678 PMC5394108

[B15] Hunt K. J. Hung S. K. Ernst E. (2010). Botanical extracts as anti-aging preparations for the skin: a systematic review. Drugs Aging 27, 973–985. 10.2165/11584420-000000000-00000 21087067

[B16] Jiang Y. Hua J. Wang B. Yuan H. Ma H. (2018). Effects of variety, season, and region on theaflavins content of fermented Chinese congou black tea. J. Food Qual. 2018, 1–9. 10.1155/2018/5427302

[B17] Li F. Dong C. X Yang T. Ma J. Zhang S.. (2019). Seasonal theanine accumulation and related gene expression in the roots and leaf buds of tea plants (*Camellia sinensis* L.). Front. Plant Sci. 10:1397. 10.3389/fpls.2019.01397 31749819 PMC6842895

[B18] Li F. Dong C. Yang T. Bao S. Fang W. Lucas W. J.. (2021). The tea plant CsLHT1 and CsLHT6 transporters take up amino acids, as a nitrogen source, from the soil of organic tea plantations. Hortic. Res. 8:178. 10.1038/s41438-021-00615-x 34333546 PMC8325676

[B19] Li F. Li H. Dong C. Yang T. Zhang S. Bao S.. (2020). Theanine transporters are involved in nitrogen deficiency response in tea plant (Camellia sinensis L.). Plant Signal Behav. 15:1728109. 10.1080/15592324.2020.1728109 32067561 PMC7194376

[B20] Ruan J. Ma L. Yang Y. (2012). Magnesium nutrition on accumulation and transport of amino acids in tea plants. J. Sci. Food Agric. 92, 1375–1383. 10.1002/jsfa.4709 22083631

[B21] Schmittgen T. D. Zakrajsek B. A. (2000). Effect of experimental treatment on housekeeping gene expression: validation by real-time, quantitative RT-PCR. J. Biochem. Biophys. Methods 46, 69–81. 10.1016/S0165-022X(00)00129-9 11086195

[B22] Sharma E. Joshi R. Gulati A. (2018). L-Theanine: An astounding sui generis integrant in tea. Food Chem. 242:601. 10.1016/j.foodchem.2017.09.046 29037735

[B23] Snowden C. J. Thomas B. Baxter C. J. Smith A. C. Sweetlove L. J. (2014). A tonoplast Glu/Asp/GABA exchanger that affects tomato fruit amino acid composition. Plant J. 81, 651–660. 10.1111/tpj.12766 25602029 PMC4950293

[B24] Song H. Zhang X. Shi C. Wang S. Wu A. Wei C. L. (2016). Selection and verification of candidate reference genes for mature microRNA expression by quantitative RT-PCR in the tea plant (*Camellia sinensis*). Genes 7:25. 10.3390/genes7060025 27240406 PMC4929424

[B25] Su Y. H. Frommer W. B. Ludewig U. (2004). Molecular and functional characterization of a family of amino acid transporters from Arabidopsis. Plant Physiol. 136, 3104–3131. 10.1104/pp.104.045278 15377779 PMC523371

[B26] Tanaka T. Watarumi S. Fujieda M. Kouno I. (2005). New black tea polyphenol having N-ethyl-2-pyrrolidinone moiety derived from tea amino acid theanine: Isolation, characterization and partial synthesis. Food Chem. 93, 81–87. 10.1016/j.foodchem.2004.09.013

[B27] Tegeder M. Tan Q. Grennan A. K. Patrick J. W. (2007). Amino acid transporter expression and localisation studies in pea (*Pisum sativum*). Funct. Plant Biol. 34, 1019–10128. 10.1071/FP07107 32689430

[B28] Wan X. (2003). Tea Biochemistry, 3rd edition. Beijing, China: China Agriculture Press (in Chinese).

[B29] Wan X. Xia T. (2015). Secondary Metabolism of Tea Plant, 1st edition. Beijing, China: Science Press (in Chinese).

[B30] Wei C. Yang H. Wang S. Zhao J. Liu C. Gao L.. (2018). Draft genome sequence of Camellia sinensis var. sinensis provides insights into the evolution of the tea genome and tea quality. Proc. Natl. Acad. Sci. USA. 115, E4151–E4158. 10.1073/pnas.1719622115 29678829 PMC5939082

[B31] Xu W. Song Q. Li D. Wan X. (2012). Discrimination of the production season of Chinese green tea by chemical analysis in combination with supervised pattern recognition. J. Agric. Food Chem. 60, 7064–7070. 10.1021/jf301340z 22720840

[B32] Yamaguchi S. Ninomiya K. (2000). Umami and food palatability. J. Nutr. 130, 921s−926s. 10.1093/jn/130.4.921S 10736353

[B33] Yang H. Bogner M. Stierhof Y. D. Ludewig U. (2010). H+-independent glutamine transport in plant root tips. PLoS ONE. 5:e8917. 10.1371/journal.pone.0008917 20111724 PMC2811748

[B34] Yang H. Krebs M. Stierhof Y. D. Ludewig U. (2014). Characterization of the putative amino acid transporter genes AtCAT2, 3 and 4: the tonoplast localized AtCAT2 regulates soluble leaf amino acids. J Plant Physiol. 171, 594–601. 10.1016/j.jplph.2013.11.012 24709150

[B35] Yang H. Stierhof Y. D. Ludewig U. (2015). The putative cationic amino acid transporter 9 is targeted to vesicles and may be involved in plant amino acid homeostasis. Front. Plant Sci. 6:212. 10.3389/fpls.2015.00212 25883600 PMC4381505

[B36] Yang T. Li H. Tai Y. Dong C. Cheng X. Xia E.. (2020). Transcriptional regulation of amino acid metabolism in response to nitrogen deficiency and nitrogen forms in tea plant root (*Camellia sinensis* L.). Sci. Rep. 10:6868. 10.1038/s41598-020-63835-6 32321966 PMC7176667

[B37] Yang Y. Yang L. Li Z. (2013). Molecular cloning and identification of a putative tomato cationic amino acid transporter-2 gene that is highly expressed in stamens. Plant Cell Tiss. Organ. 112, 55–63. 10.1007/s11240-012-0215-9

[B38] Yao L. Liu X. Jiang Y. Caffin N. Arcy B. D. Singanusong R.. (2006). Compositional analysis of teas from Australian supermarkets. Food Chem. 94, 115–122. 10.1016/j.foodchem.2004.11.009

[B39] Yoneda Y. (2017). An L-glutamine transporter isoform for neurogenesis facilitated by L-theanine. Neurocheml Res. 42, 2686–2697. 10.1007/s11064-017-2317-6 28597057

[B40] Zeng L. Zhou X. Liao Y. Yang Z. (2020). Roles of specialized metabolites in biological function and environmental adaptability of tea plant (*Camellia sinensis*) as a metabolite studying model. J. Adv. Res. 21:65. 10.1016/j.jare.2020.11.004 35024188 PMC8655122

[B41] Zhu B. Y. Guo J. Y. Dong C. X. Li F. Qiao S. M. Lin S. J.. (2021). CsAlaDC and CsTSI work coordinately to determine theanine biosynthesis in tea plants (*Camellia sinensis* L.) and confer high levels of L-theanine accumulation in a non-tea plant. Plant Biotechnol. J. 10.1111/pbi.13722 34626137 PMC8633503

