# Supplementary figures and images for: Tonoplast-Localized Theanine Transporter CsCAT2 May Mediate Theanine Storage in the Root of Tea Plants (Camellia sinensis L.)
Source: Front Plant Sci. 2021 Dec 17;12:797854. doi: 10.3389/fpls.2021.797854 (PMC8719441; doi:10.3389/fpls.2021.797854)

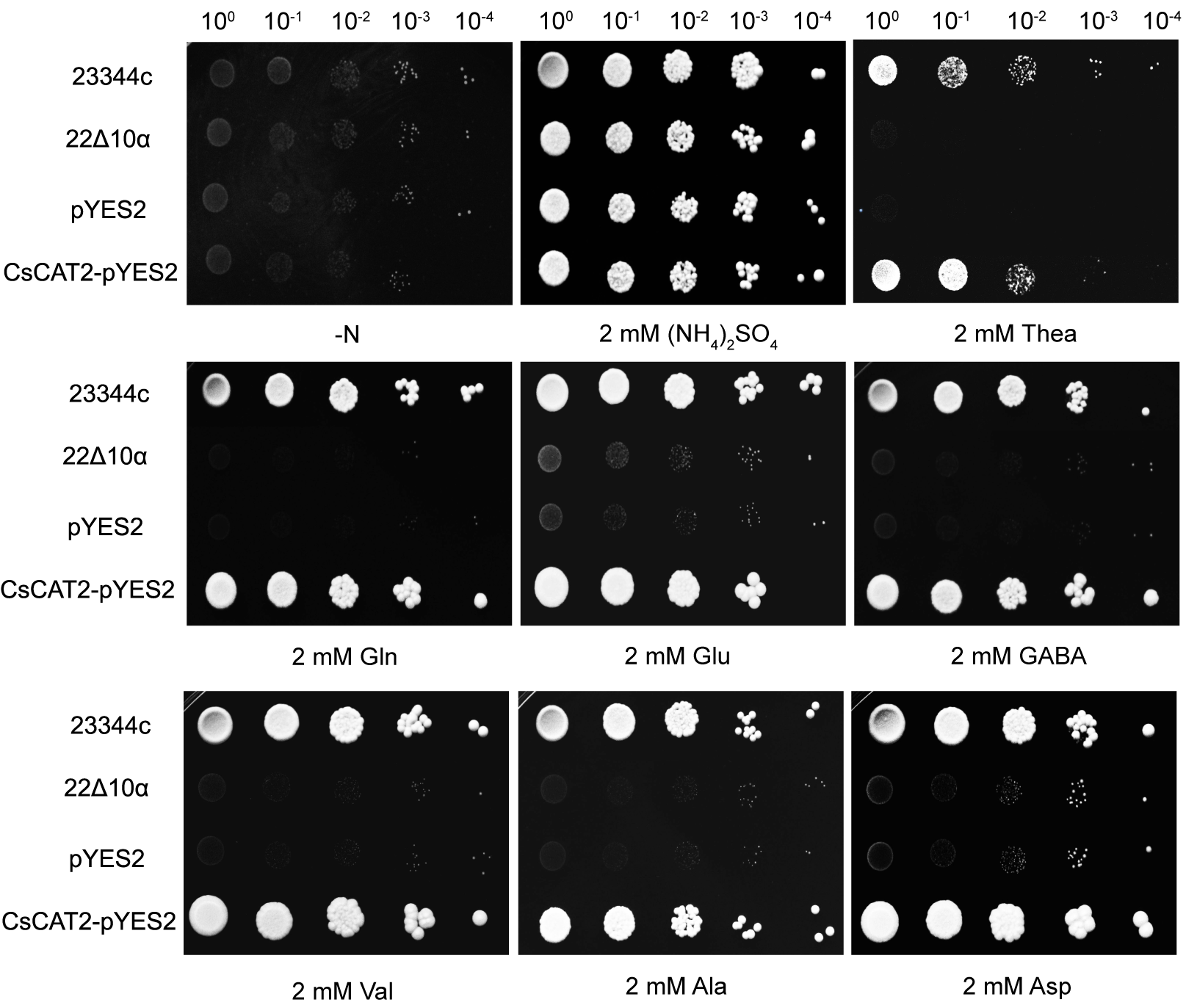

Supplement: Supplementary Figure 1 — Growth of yeast strains on medium with amino acid as sole nitrogen source, with 0 nitrogen (-N) and 2 mM (NH4)2SO4 as negative and positive controls. [file Image_1.TIF]
