# Supplementary material for: Tonoplast-Localized Theanine Transporter CsCAT2 May Mediate Theanine Storage in the Root of Tea Plants (Camellia sinensis L.)
Source: Front Plant Sci. 2021 Dec 17;12:797854. doi: 10.3389/fpls.2021.797854 (PMC8719441; doi:10.3389/fpls.2021.797854)
Supplement: Supplementary Table 1 — Primers used in the experiments. [file Table_1.DOC]

**Supplementary Table S1. Primers used in the experiments.**

| Primer name | Gene name | | | | Primer sequence 5’-3’ |
| --- | --- | --- | --- | --- | --- |
| Primers for Gene Cloning in Yeast | | | | | |
| *CsCAT2-F* | *CsCAT2* | | CCGCTCGAGCATGCATCTAGAATGGGTTTTCGTTGTGATTCACAAAAT | | |
| *CsCAT2-R* |  |  | TACATGATGCGGCCCTCTAGACTAAGGTAGAGAGTTTTCAGAGCTCC | | |
| Primers for RT-PCR used in this study | | | | | |
| *CsCAT2-RT-F* | *CsCAT2* | | TCCTGATACTCCCATTTCTTCTGC | | |
| *CsCAT2-RT-R* |  |  | GCTCTTGATAGGAACTTGGGTTCGTTT | | |
| *GAPDH-F* | GAPDH | | TTGGCATCGTTGAGGGTCT | | |
| *GAPDH-R* |  |  | CAGTGGGAACACGGAAAGC | | |
| Primers design for Transient Expression in protoplasts | | | | | |
| *GateCsCAT2-F* | | *CsCAT2* | | GGGGACAAGTTTGTACAAAAAAGCAGGCTTCATGGGTTTTCGTTGTGATTCACAAAAT | |
| *GateCsCAT2-R* | |  |  | GGGGACCACTTTGTACAAGAAAGCTGGGTCTAAGGTAGAGAGTTTTCAGAGCTCC | |
